# Age-related changes in ultrasound-assessed muscle composition and postural stability

**DOI:** 10.1038/s41598-024-69374-8

**Published:** 2024-08-12

**Authors:** Scott J. Mongold, Christian Georgiev, Gilles Naeije, Marc Vander Ghinst, Matt S. Stock, Mathieu Bourguignon

**Affiliations:** 1 Laboratory of Neurophysiology and Movement Biomechanics, UNI–ULB Neuroscience Institute Université libre de Bruxelles (ULB), 1070 Brussels, Belgium; 2 Laboratoire de Neuroanatomie et Neuroimagerie Translationnelles, UNI–ULB Neuroscience Institute, Université libre de Bruxelles (ULB), 1070 Brussels, Belgium; 3 Centre de Référence Neuromusculaire, Department of Neurology, CUB Hôpital Erasme, Université libre de Bruxelles (ULB), 1070 Brussels, Belgium; 4 Service d’ORL et de Chirurgie Cervico-Faciale, CUB Hôpital Erasme, Université libre de Bruxelles (ULB), 1070 Brussels, Belgium; 5 School of Kinesiology and Rehabilitation Sciences, University of Central Florida, Orlando, Florida, 32816 USA; 6 BCBL, Basque Center on Cognition, Brain and Language, 20009 San Sebastian, Spain

**Keywords:** Ageing, Muscle, Ultrasonography

## Abstract

While the simultaneous degradation of muscle composition and postural stability in aging are independently highly investigated due to their association with fall risk, the interplay between the two has received little attention. Thus, the purpose of this study is to explore how age-related changes in muscle composition relate to postural stability. To that aim, we collected posturography measures and ultrasound images of the dominant Vastus Lateralis and Biceps Brachii from 32 young (18–35 year old) and 34 older (65–85 year old) participants. Muscle properties were quantified with echo-intensity and texture-based metrics derived from gray-level co-occurrence matrix analysis, and postural stability with the variability of the center of pressure during bipedal stance tasks. Ultrasound parameters revealed that young muscle possessed lower echo-intensity and higher homogeneity compared to the elderly. Echo-intensity and muscle thickness, and several texture-based parameters possessed outstanding young versus older classification performance. A canonical correlation analysis demonstrated a significant relationship between ultrasound and postural measures only within the young group (*r* = 0.53, *p* < 0.002), where those with ‘better’ muscle composition displayed larger postural sways. Our results indicate that, in older individuals, postural stability and muscle composition, two common fall risk factors, are unrelated. In view of this decoupling, both may contribute independently to fall risk. Furthermore, our data support the view that texture-based parameters provide a robust alternative to echo-intensity in providing markers of muscle composition.

## Introduction

Aging seems to be marked by unavoidable physical decline; however, by investigating the underlying mechanisms of aging, we can challenge the notions of what it means to grow older. For many elderly, aging results in an increased incidence of falls, with deleterious consequences for the elderly themselves^1–3^, and for society^4^. Unfortunately, the underpinnings of falls and poor balance are unresolved.

Marked differences in postural stability have been identified between young and elderly groups^5,6^, as well as between fallers and non-fallers^7,8^. Notably, elderly people tend to lack stability across both anterior–posterior and medio-lateral directions during standing balance tasks^9,10^.

Recent evidence in older adults suggests that muscle composition may play a role in fall risk. Indeed, increased intramuscular fat was related to decreased ability to perform a biomechanically stable protective step in balance recovery^11^, gluteal muscle composition could differentiate fallers from non-fallers^12^, and hip intramuscular fat was associated with increased gait variability^13^. Although alterations in muscle properties and postural stability appear to be implicitly linked to the cause of falls in the elderly, their dependence is poorly documented. Yet, some possible underlying mechanisms have been suggested, according to which increased intramuscular fat could impair neuromuscular activation^11^, or indirectly alter proprioception through mechanical changes to muscles^14^.

Ultrasonography (US) represents an alternative, accessible muscle imaging tool, capable of assessing both muscle size and composition, with the benefit of timeliness, cost-effectiveness, and reduced risk, as compared to dual x-ray absorptiometry and magnetic resonance imaging. Importantly, US measures have been shown to be related to many functional measures, including muscle strength^15^, power^16,17^, and even cardiorespiratory fitness^17^. While these functions remain essential to maintaining independence, the relationship between US measures and standing stability is not well studied.

Notwithstanding, US imaging comes with the caveat that conventional US echo-intensity (EI) analyses have been shown to depend on equipment settings like gain and time-gain compensation^18^. However, these limitations may be circumvented by texture-based analyses that have been shown to be robust to changes in US equipment settings^18,19^. The gray-level co-occurrence matrix (GLCM) statistical measures were developed for statistical 2D textural analysis^20^ and are the most commonly employed texture-based analysis method. This second order approach can be generally defined as the mathematical characterization of pixel intensities and their spatial distribution within a region of interest (ROI)^21^. Interestingly, there is some evidence that the application of texture analyses to musculoskeletal US images is sensitive to both pathology^22,23^ and age^19,24^. In this context, higher homogeneity is indicative of denser muscle tissue, and accordingly, younger individuals tend to possess muscles that are more homogenous compared to their older counterparts^19,24^. And while more studies are needed to fully support these findings, texture-based parameters represent an interesting addition to studies featuring US muscle assessment.

The present study assessed the link between the composition of muscle, in addition to its size, and standing stability across several balance conditions to determine whether muscle composition relates to postural performance and how this is impacted by age. The specific goals of this study are to (i) investigate the influence of age on skeletal muscle assessed with conventional EI and texture-based parameters, (ii) assess the ability of these parameters to differentiate young and older muscle, and (iii) determine how they jointly relate to postural performance. Previous work has shown that the upper and lower extremity respond disparately during aging^25,26^. Therefore, we assessed muscle morphology and composition in one lower and upper body muscle, the Vastus Lateralis (VL) and Biceps Brachii (BB), both of which were selected because of the superficial location and image reliability^27,28^. We assessed postural performance across a range of conditions, with vision and/or standing surface altered. We expected better muscle composition—as assessed by EI and texture-based parameters—and standing stability in younger participants and that more favorable muscle composition would be associated with better upright standing stability. Ultimately, this research should contribute to better understanding the causes of postural instability in the elderly, and help guide future research and interventions aimed at limiting fall risk.

## Methods

### Participants

Sixty-six healthy participants took part in this study. Of them, 32 formed the young group (15 female; mean ± SD age, 25.5 ± 2.9 years; range 19–31 years) and 34 formed the older group (18 female; mean ± SD age, 72.1 ± 6.0 years; range 65–85 years). Participants reported no history of neuromuscular disorders, orthopedic limitation, or balance-related disorders or medications affecting balance, and were self-reported as generally healthy. No older participants had sarcopenia diagnoses.

Table 1 presents participants’ demographics. Significant differences were found in average age and height, but not in sex ratio (*p* = 0.81, Fisher exact test), weight or BMI.

**Table 1:** Physical characteristics of all participants.

Variables	Young group (n = 32)	Old group (n = 34)	*p*-value	*t*_64_
Number and proportion of women	15, 46.8%	18, 52.9%	0.81	N/A
Age (years)	25.5 ± 2.9	72.1 ± 6.0	< 0.0001	− 40.1
Height (cm)	173.0 ± 10.0	166.4 ± 9.4	0.008	2.75
Weight (kg)	74.8 ± 14.6	72.0 ± 12.2	0.412	0.825
BMI (kg/m^2^)	25 ± 4.4	26 ± 4.1	0.315	− 1.01

Data presented as mean ± SD, unless otherwise stated.

Participants were asked not to exercise within 48 h of the experiment and follow their typical sleep behavior. The experimental protocol was approved by the ethics committee of the Université libre de Bruxelles. All participants gave written informed consent in accordance with the Declaration of Helsinki.

### Experimental protocol

All participants completed posturography measures, followed by US imaging of their dominant VL and BB.

### Posturography

**Figure 1:**
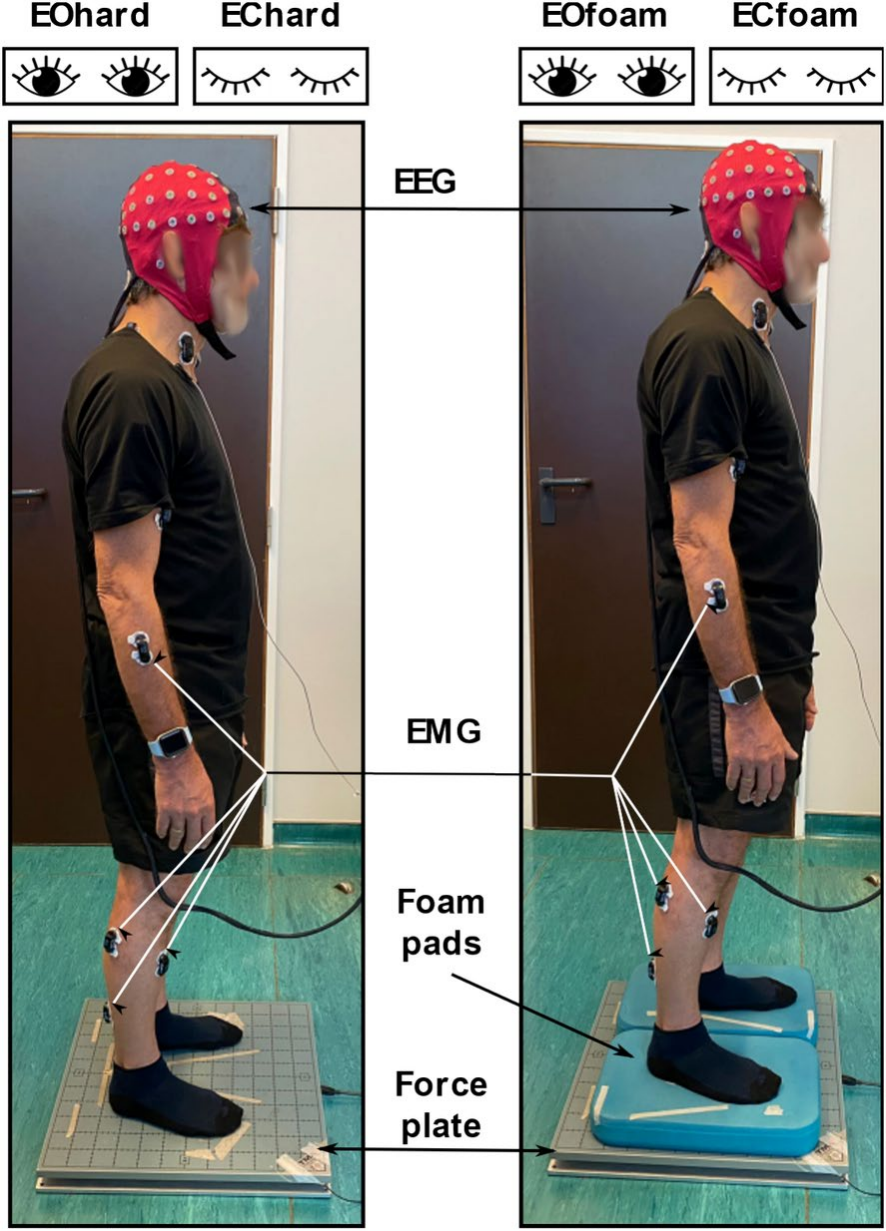
Experimental set-up. Participants were equipped with a 64 channel EEG-cap, 15 bilateral EMG sensors, and stood on a force plate in 4 experimental conditions: either on a hard surface or on foam pads, and with eyes open or closed. EEG and EMG were not analyzed for this study.

Participants completed 2 randomized blocks of posturography, each consisting of 4 conditions. Participants were asked to stand upright, keep their arms at their sides, and maintain their balance. Each condition lasted for 5 min. Participants were asked to stand on a force plate with eyes open (EOhard) or eyes closed (EChard), and on the force plate, but with foam pads under each foot (Domyos, Decathlon, Villeneuve-d’Ascq, France) with eyes open (EOfoam) or eyes closed (ECfoam). Previous work has identified differences in stability across the mentioned condition types, establishing that older individuals tend to sway more with eyes closed and on compliant surfaces^6,7^. Their feet were oriented in a comfortable position and spaced at approximately shoulder width. A red cross was placed approximately 1.5 m in front of the participant at eye level. This target served as a fixation point for each participant to encourage minimal movement of the head and shoulders over the course of each condition. Ground reaction forces and moments were recorded at 1000 Hz during each condition with the force plate (AccuSway-O, AMTI, Watertown, MA, USA). Figure 1 depicts the posturography set-up.

### Ultrasonography

Participants sat for approximately 15 min to stabilize fluid shifts that might affect our measures, as done previously^29,30^. The Vscan Air (GE Healthcare, USA), a 3rd generation point-of-care wireless US device was used to conduct B-mode sonography. This is a two-headed probe with a convex and linear side. For this study, the linear side of the probe was used. Linear probe specifications include a broad-bandwidth linear array: from 3 to 12 MHz, with dimensions 131 × 64 × 31 mm, and imaging depth up to 8 cm. For all acquisitions, the US images were visualized on the same smartphone (iPhone 13, Apple, USA) using the Vscan Air application (freely available on Apple or Google app stores). Imaging settings within the application remained the same for all participants: gain was set to 40% and image depth was set to 5 cm. One trained experimenter (SJM) with extensive US experience conducted the imaging.

Two anatomical sites were selected for US imaging, the VL and BB, as two important muscles in locomotion and upper extremity function, respectively. Three images were acquired from each participant and each muscle, after having applied US gel. The transducer was oriented transverse to the segment, approximately 10 cm from the superior edge of the patella for the VL, and at the midpoint between the axilla and elbow for the BB. Images were captured and stored within the Vscan Air application and later exported in .jpg format to be analyzed offline.

### Force plate data processing

All force plate data was processed with custom scripts in Matlab (Mathworks, Natick, MA, USA). Center of pressure (COP) was calculated using the raw data from the force moments and ground reaction force from the force plate. COP time-series were filtered between 0.1 and 10 Hz. Postural stability was quantified as the standard deviation of COP (sdCOP) along the anterior–posterior (sdCOP_AP_) and medio-lateral axes (sdCOP_ML_), so that low sdCOP indicated good stability and high sdCOP indicated poor stability. A recent systematic review described that postural sway differences were most evident in the anterior–posterior^10^, so we will mainly focus on sdCOP_AP_ and present results for sdCOP_ML_ in Supplementary Materials.

### US image processing

**Figure 2:**
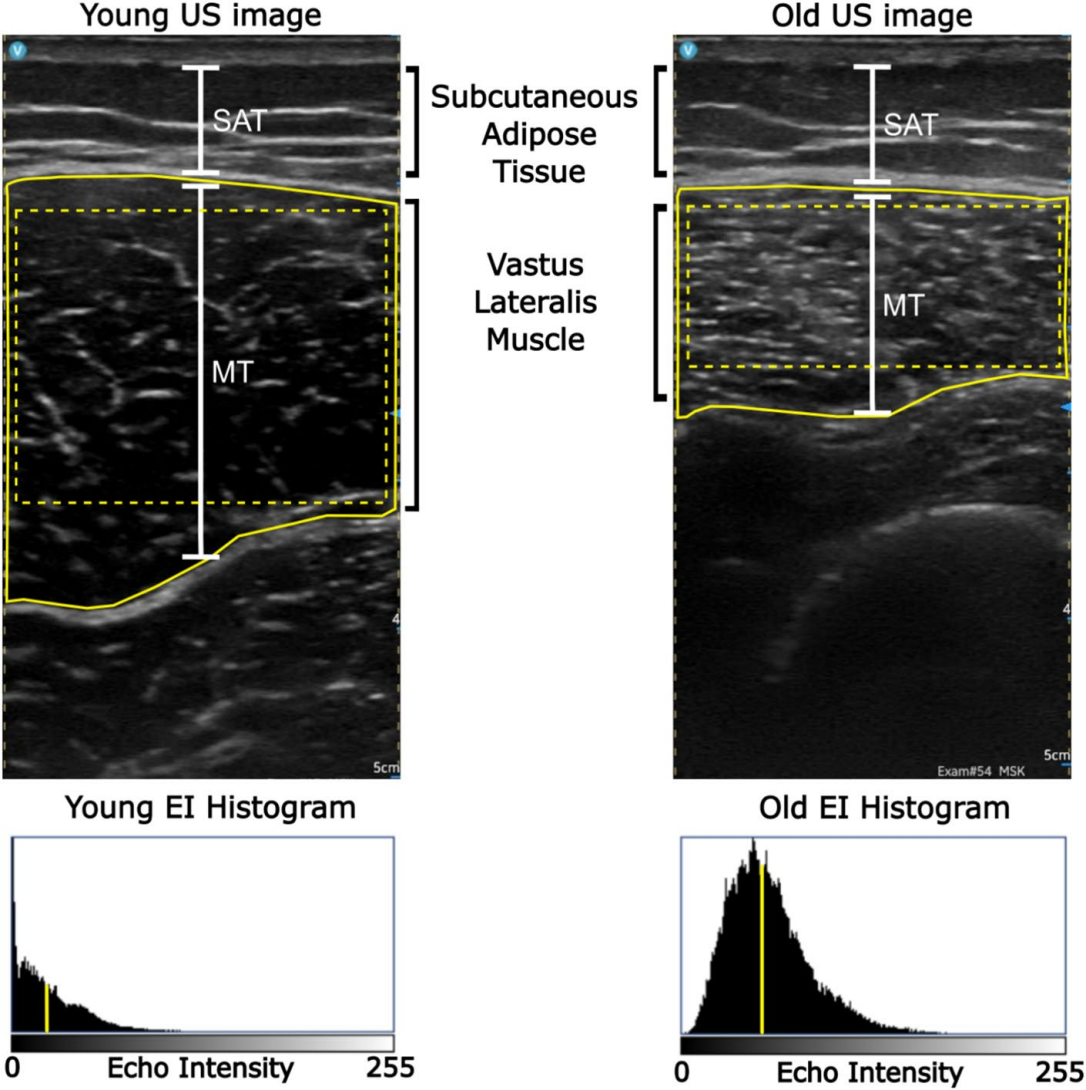
Processing of thigh-segment US images. US image of young female shown on left and US image of an older female shown on the right. Vertical white lines represent estimation of muscle thickness (MT) and subcutaneous adipose thickness (SAT). A polygon (in solid yellow) outlines the entire muscle cross-section, which served as the ROI for EI analysis. A rectangle (in dashed yellow) outlines the ROI for texture analysis. Below each US image is the resultant EI histogram. Vertical yellow line indicates mean pixel intensity.

The US images were analyzed with ImageJ software (National Institutes of Health, Bethesda, MD). The muscle thickness (MT) of the VL and BB was estimated as the distance between the superficial and deep aponeurosis for the VL and superficial aponeurosis and the humerus for the BB in the center of each image (see Fig. 2 for VL example), as done previously^30^. Subcutaneous adipose thickness (SAT) at both muscle locations was measured as the distance between the skin and the border of the superficial aponeurosis in the center of each image (see Fig. 2), akin to previous work^30,31^. For EI assessment, the ROI was the entire cross sectional area of each muscle of interest, which was outlined manually with a polygon (see Fig. 2). Care was taken not to include aponeurosis or bone within the ROI. The EI value was computed as the mean pixel intensity across the ROI. Pixel intensity ranged from 0 (black) to 255 (white) on an arbitrary scale maintained constant across all participants. MT, SAT, and EI measures were assessed on the three different images at each location, and their average value is reported. Often, EI is corrected by SAT; however there were no differences between the younger and older group SAT measures, therefor the raw EI values are reported. The same experimenter (SJM) conducted all of the analysis and was blind to participant age.

A texture-based image analysis was employed to investigate the spatial variation in pixel intensity of both muscles. ImageJ was used for the analysis. The GLCM characteristics were explored using a plugin macro (https://imagej.nih.gov/ij/plugins/texture.html, Texture Analyzer v0.4, Julio E. Cabrera) for ImageJ. This plugin requires rectangle-shaped ROIs for the GLCM analysis, which is why such ROIs were created, with the goal of incorporating as much muscle as possible without including aponeurosis or bone (see Fig. 2). Τhe GLCM computation was performed in four directions (0°, 90°, 180° and 270°), and the resultant values were averaged to mitigate the effect of direction^32,33^. Outcome parameters were the angular second moment (ASM), contrast, correlation, inverse different moment (IDM), and entropy. We followed the approach of previous works using 5 of the 14 total Haralick features, as high correlations between features have been observed^34^. The selected Haralick features are thought to summarize important information about the textural arrangement of images^19,32^. ASM, sometimes referred to as energy, assesses gray-level uniformity, or homogeneity. Contrast measures the variation in intensity between a pixel and its neighbors. Correlation measures the level of linear predictability between adjacent pairs. IDM measures the difference between adjacent pairs, and increases as these differences become smaller. Entropy quantifies textural disorder, or the distribution of adjacent pair combinations, increasing as more occupied adjacent pair combinations are observed. For a more extensive description of these parameters, we refer the reader to the article by Wilkinson et al.^32^. Overall, increased ASM, correlation, and IDM values indicate high homogeneity within an image, and increased contrast and entropy indicate high heterogeneity within an image. Mathematical formulas for each parameter can be found in Table 1 of a recently published related work^33^. Reported parameters were averaged across the 3 images at each location.

### Statistical analysis

Statistical analyses were performed using Matlab and SPSS (v25, IBM, Armonk, NY, USA).

Intraclass correlation coefficients (ICC) and their 95% confidence interval (95% CI) were computed to assess test–retest reliability of the US measures across all parameters. Reliability is considered low for ICC ≤ 0.50, moderate for 0.50 < ICC ≤ 0.75, good for 0.75 < ICC ≤ 0.9 and excellent for ICC ≥ 0.90.

Between group differences in US parameters were assessed using Independent samples t-tests.

A receiver operating characteristic (ROC) analysis was used to estimate the ability of US parameters to classify participants as young or older. This analysis was carried out with VL measures only, in view of the direct implication of this muscle (but not of the BB) in regulating balance. Using empirical (non-parametric) curve estimation as described previously^35^, a z-test was used for comparing the AUC of two ROCs at a time. Measures that produced an area under the curve (AUC) above 0.50 indicate that the classifier performs above chance-level in discriminating between young and older muscle. Previous work describes AUCs between 0.7 and 0.8 as acceptable, between 0.8 and 0.9 as excellent, and above 0.9 as outstanding^36^.

A two-way ANOVA was used to examine differences in postural stability across the four conditions and age groups. In this analysis, the dependent variable was the sdCOP normalized by participants height. Student t-tests with Bonferroni correction for multiple comparisons were used for post-hoc analyses.

A canonical correlation analysis (CCA) was used to assess the relationship between US parameters and postural stability across the four postural conditions. CCA is often used when there are multiple intercorrelated variables. Variables were standardized, corrected for outliers greater or less than 2.5 standard deviations from the mean, and corrected for age by linear regression. Outliers less than the minimum value were replaced with the minimum value and outliers greater than the maximum value were replaced with the maximum value. Regularization was used to deal with the limited sample size at our disposition^37^, with regularization parameters selected through leave-one-out cross-validation. The statistical significance of the final regression model was assessed with permutation statistics, by comparing the correlation value to its permutation distribution (1,000 permutations) obtained after having shuffled US parameters—but not postural stability—across participants. Further correlation analysis was carried out using the Pearson correlation coefficient, assessing the relationship between the pooled parameters. Pooling was done as informed by CCA weights. The correlation value was qualitatively assessed as follows: 0.00–0.19, very weak; 0.20–0.39, weak; 0.40–0.59, moderate; 0.60–0.79, strong; 0.80–1, very strong^38^.

## Results

### VL muscle morphology, EI, and GLCM measures

Table 2 presents the characteristics of the VL muscle in the young and older groups and their statistical comparisons. Conventional US metrics (MT, EI, and SAT) showed that younger participants tended to possess larger muscles, with less intramuscular fat/connective tissue distributed throughout the cross-section, as compared to their older counterparts. Specifically, MT in the VL was on average ~ 50% larger in young compared to older participants and its EI was ~ 40% lower. However, SAT over the VL muscle belly was similar between groups.

**Table 2:** VL muscle characteristics of all participants.

Variables	Young group	Older group	*p*-value	t_64_	Cohen’s d	ICC 95% CI
MT (cm)	2.21 ± 0.48	1.38 ± 0.33	< 0.0001	8.22	2.0	0.97–0.99
EI (A.U.)	32.3 ± 7.1	56.0 ± 9.2	< 0.0001	− 11.6	2.9	0.95–0.98
SAT (cm)	0.98 ± 0.48	0.93 ± 0.49	0.72	0.354	0.1	0.94–0.99
Texture parameters						
ASM	0.008 ± 0.02	0.001 ± 0.0003	0.005	2.89	0.7	0.97–0.99
Contrast	66.9 ± 25.9	86.1 ± 30.6	0.008	− 2.73	0.7	0.92–0.99
Correlation	0.001 ± .0006	0.001 ± 0.0004	0.26	1.13	0.3	0.90–0.99
IDM	0.295 ± 0.06	0.224 ± 0.03	< 0.0001	6.63	1.6	0.91–0.99
Entropy	7.17 ± 0.50	7.77 ± 0.31	< 0.0001	− 5.87	1.4	0.87–0.99

Data presented as mean ± SD.

GLCM measures in the VL showed significant differences between the young and older groups, in all parameters except Correlation. ASM and IDM were higher in young compared to older participants, and vice-versa for Contrast and Entropy (Table 2), indicating higher heterogeneity in older participants. Indeed, higher values of ASM, Correlation, and IDM correspond to images with higher homogeneity, whereas higher values of Contrast and Entropy denote images with higher heterogeneity.

ICC analysis showed high reliability across participants for all VL-related parameters.

### BB muscle morphology, EI, and GLCM measures

Table 3 presents the characteristics of the BB muscle in the young and older groups and their statistical comparisons. BB muscle MT and SAT did not differ between the young and older groups. Similar to the VL, the EI of the BB was lower in the younger group, by ~ 30%.

**Table 3:** BB muscle characteristics of all participants.

Variables	Young group	Older group	*p*-value	t_64_	Cohen’s d	ICC 95% CI
MT (cm)	2.79 ± 0.65	2.68 ± 0.66	0.47	0.722	0.2	0.98–0.99
EI (A.U.)	30.0 ± 6.9	42.3 ± 8.4	< 0.0001	− 6.48	1.6	0.91–0.96
SAT (cm)	0.52 ± 0.28	0.60 ± 0.29	0.22	− 1.24	0.3	0.83–0.99
Texture parameters						
ASM	0.013 ± 0.02	0.001 ± 0.002	0.006	2.82	0.7	0.96–0.99
Contrast	119.1 ± 45.8	138.6 ± 59.0	0.14	− 1.49	0.4	0.85–0.99
Correlation	0.001 ± 0.0004	0.0008 ± 0.0001	0.02	2.47	0.6	0.74–0.99
IDM	0.249 ± 0.07	0.176 ± 0.04	< 0.0001	5.16	1.3	0.86–0.99
Entropy	7.33 ± 0.66	8.09 ± 0.39	< 0.0001	− 5.69	1.4	0.85–0.99

Data presented as mean ± SD.

GLCM measures in the BB showed significant differences between young and older groups, in all parameters except Contrast. Again similar to the VL, differences in all other 4 GLCM measures were indicative of higher heterogeneity in older compared with young groups.

ICC analysis showed high reliability across participants for all BB-related parameters.

### Classification analysis

**Figure 3:**
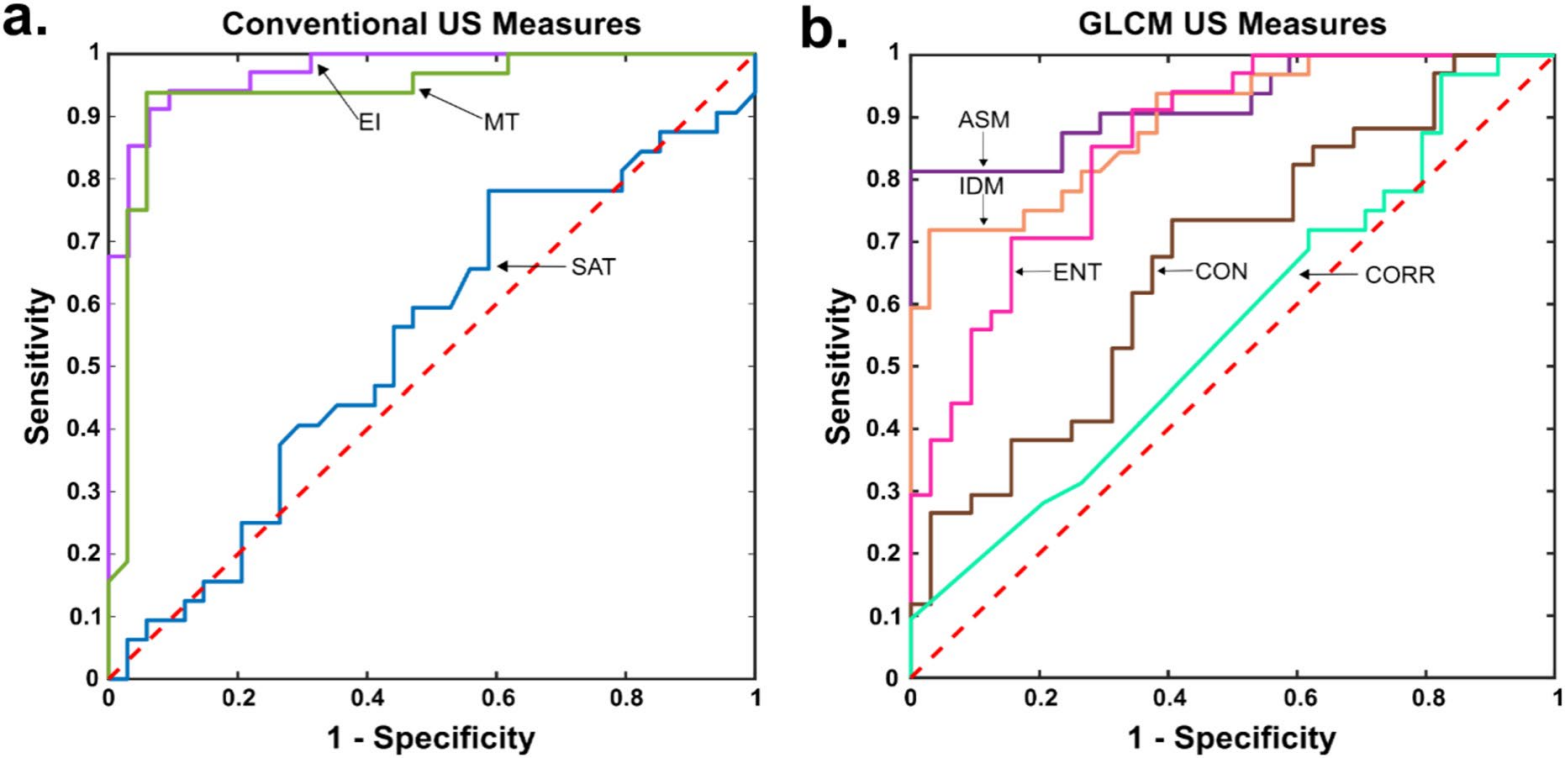
ROC curves for conventional US parameters (**a**) and texture-based parameters (**b**). The dotted red line corresponds to the performance of a random classifier. *ENT* Entropy; *CON* Contrast; *CORR* Correlation.

Figure 3 shows the resultant ROC curves for all measures, separated as conventional (Fig. 3a) and texture-based (Fig. 3b). Two conventional parameters had an AUC corresponding to outstanding classification performance: EI (AUC = 0.972) and MT (AUC = 0.936), while SAT had an AUC indicating classification was nearly random (AUC = 0.540). For texture-based parameters, ASM demonstrated outstanding classification performance (AUC = 0.924), and both IDM (AUC = 0.895) and Entropy (AUC = 0.857) performed excellently. Contrast (AUC = 0.671) and Correlation (AUC = 0.563) did not possess robust classification performance.

The classification ability amongst parameters was assessed by comparing AUCs. For the conventional parameters, the AUC for EI was no different from that for MT (*z* = 1.30, *p* = 0.195) indicating similar performance, but both were significantly greater compared to that for SAT (EI, *z* = 5.77, *p* < 0.0001; MT, *z* = 4.53, *p* < 0.0001).

Similar comparisons were made for the GLCM parameters. ASM and IDM performed comparably (*z* = 1.60, *p* = 0.111), and significantly better compared to all other GLCM parameters (*z* = 2.32–6.25, 0.0001 < *p* < 0.02). The classification performance of Entropy was significantly better than that of Contrast (*z* = 4.82, *p* < 0.0001) and Correlation (*z* = 6.58, *p* < 0.0001). Lastly, Contrast performed significantly better than Correlation (*z* = 3.40, *p* = 0.001).

Further comparisons of AUC between the most promising conventional and GLCM parameters revealed that EI did not perform significantly better than ASM (*z* = 1.78, *p* = 0.075), but significantly better than IDM (*z* = 2.36, *p* = 0.018). Classification performance between MT and both ASM and IDM showed no differences (*z* = 0.318, *p* = 0.750; *z* = 1.02, *p* = 0.308).

### Posturography analysis

**Figure 4:**
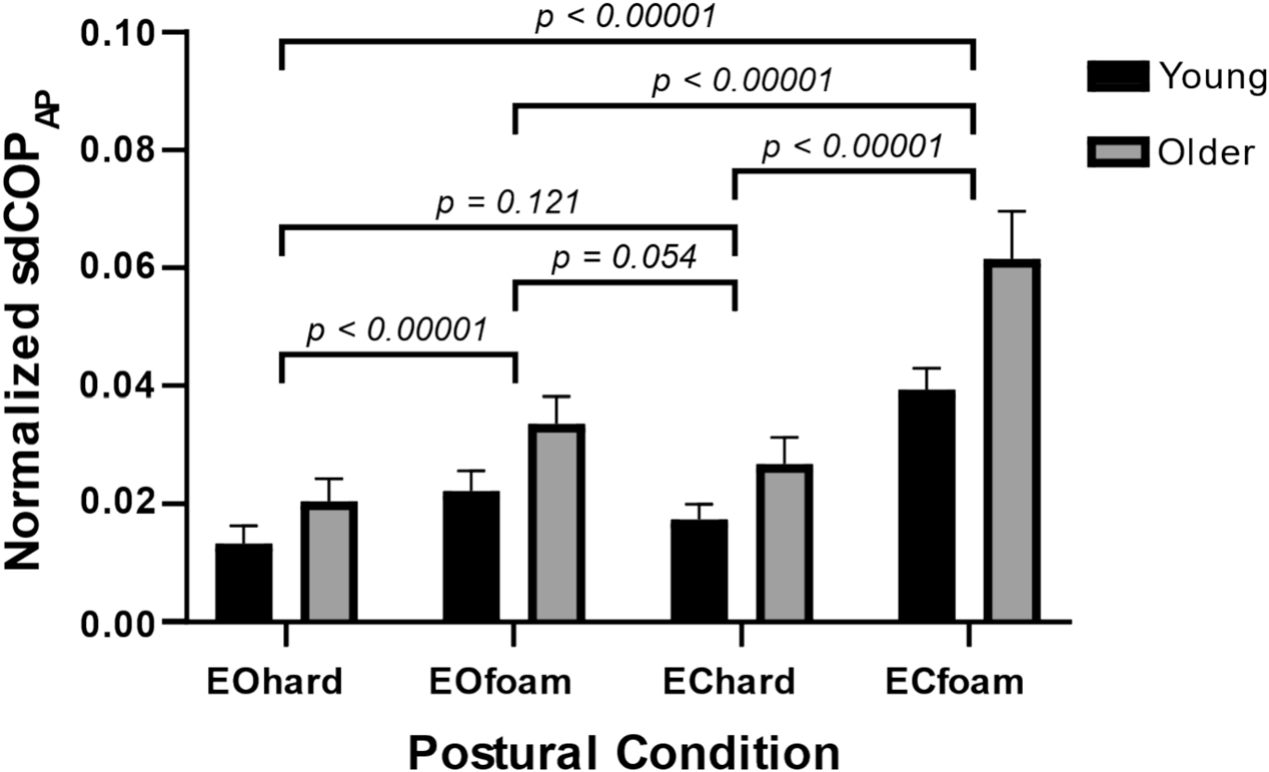
Effect of condition and age on postural stability. Vertical bars and error bars indicate the mean and standard error of the sdCOP_AP_ for each condition and group (black bars, young group; gray bars, older group). Horizontal lines indicate the *p*-value for the comparisons of normalized sdCOP_AP_ between conditions.

Figure 4 presents the sdCOP_AP_ normalized by height in both groups and in the four standing balance conditions. A two-way ANOVA applied to that measure revealed a significant effect of condition (*F*_3,263_ = 87.2, *p* < 0.0001, *η*^2^ = 0.505) and age (*F*_1,263_ = 63.0, *p* < 0.0001, *η*^2^ = 0.197), and a significant interaction thereof (*F*_3,263_ = 4.44, *p* < 0.005, *η*^2^ = 0.049). The outcome of post-hoc analyses is presented in Fig. 4. The ECfoam condition resulted in significantly higher sdCOP_AP_ compared to all other conditions, and the EOfoam condition resulted in higher sdCOP_AP_ compared to the EOhard condition. In all conditions, older participants had significantly increased sdCOP_AP_ compared to young participants (*p* < 0.005), with the greatest difference between groups in the ECfoam condition, where older participants were approximately 57% more unstable than their younger counterparts.

Similar observations of sdCOP_ML_ were obtained in relation to conditions and age groups (see Supplementary Material).

The US parameters with the strongest AUCs were included in the posturography canonical correlation analysis: EI, MT, ASM, IDM, and Entropy. The CCA was conducted on young participants and older participants separately, comparing US parameters and postural stability across the four conditions.

**Figure 5:**
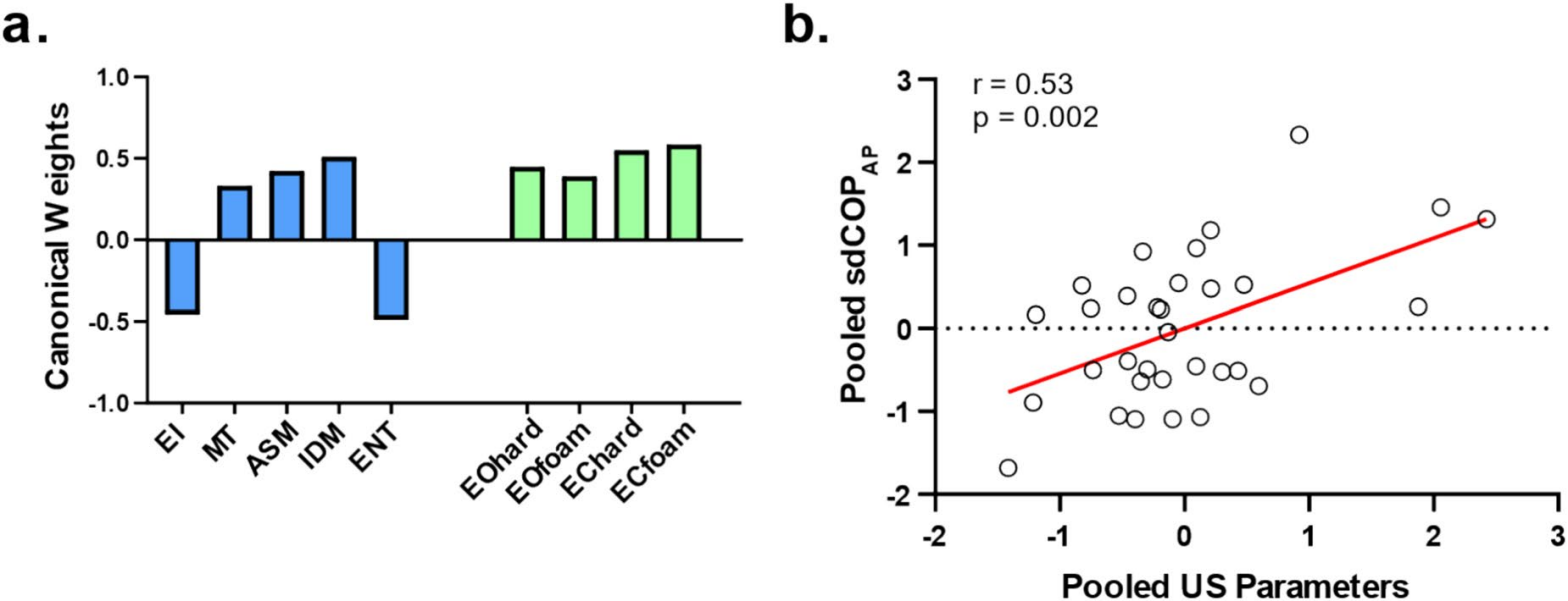
Association between US-derived muscle properties and postural sway (sdCOP_AP_). (**a**) Weights of the standardized US parameters (blue) and standardized sdCOP_AP_ in the 4 conditions (green). (**b**) Pooled standardized sdCOP_AP_ across conditions as function of pooled standardized US parameters. Circles indicate individual values, and their linear regression line is in red. Correlation value and associated significance level are indicated in the top left corner. *ENT* Entropy.

Figure 5 presents the results for the young group, where the CCA identified a significant relationship (*p* = 0.02). The CCA weights for the standardized US parameters were of similar magnitude, but varied in sign, being positive for variables indicating favorable muscle composition (MT, ASM, and IDM) and negative for those indicating unfavorable muscle quality (EI and Entropy) (Fig. 5a). Likewise, the CCA weights for standardized sdCOP_AP_ were of similar amplitude and of the same sign for the 4 conditions. Accordingly, a correlation analysis indicated a moderate relationship between the pooled US parameters (mean of the standardized parameters corrected for sign as indicated by CCA weights) and the pooled sdCOP across conditions (mean of standardized sdCOP_AP_) (Fig. 5b).

The CCA conducted on older participants did not result in a significant relationship (*p* = 0.764).

The CCA conducted on sdCOP_ML_ did not uncover any significant associations (see Supplementary material).

## Discussion

We show that with age, VL and BB muscles tend to show characteristics of poor muscle composition, with US parameters identifying increased EI and heterogeneity, in addition to reduced muscle thickness (in the VL) in older participants. As a result, both conventional and texture-based US parameters could reliably classify young and older VL muscle, providing a potential diagnostic tool for future use. Similarly, we demonstrate that elderly participants possess increased postural instability compared to young adults. However, muscle properties and standing stability were not significantly associated within older participants, while better muscle characteristics were associated with decreased stability within young participants.

Importantly, our data are aligned with previous works showing that standing balance is more unstable in older, compared to younger adults^9,10,39^. Additionally, the US measures appeared to be similar to that of previous age-related studies^15^. Altogether, it appears that the current findings should be generalizable to young and older muscle datasets.

Our results highlight age-related deterioration in muscle composition and postural stability, but these two variables were not significantly related within the older group. Therefore, physiological drivers, other than loss of muscle size and changes in composition, may be the primary cause of age-related changes in balance function. Indeed, several age-related neural changes, both peripherally and centrally have the potential to modulate balance function. For example, previous work suggests that age-induced reduction in postural stability receives contributions from decreased coordination of muscle activity^40^, alteration in sensory integration^41^, or potential changes in the processing of somatosensory afferents^42^. Importantly, our data suggests that muscle composition and balance stability, two factors commonly reported to predict fall risks^12,43,44^, are independent predictors of such risks. It could be hypothesized that upright balance stability contributes to limiting the number of events leading to excess postural sway, and thus fall risk. On the other hand, muscle composition and size would then provide the needed resources to overcome adverse events when fall-inducing sways occur^45,46^. Future work, examining additional contributors to balance function and a more heterogeneous sample of elderly participants, i.e. sedentary and active, or fallers versus non-fallers, may help further clarify the relationship between muscle composition and postural stability in aging.

In young participants, we identified a surprising relationship between muscle composition and postural parameters. Participants with larger muscles and ‘better’ muscle composition, as quantified by decreased fatty infiltration (low EI and low Entropy), and increased homogeneity (increased ASM and IDM measures), tended to be more unstable. This result is in stark opposition with our hypothesis positing that better muscle composition would be associated with more stable balance. Although this reversed relationship is puzzling, previous work suggests we may have captured an effect of training status and/or muscle fiber type composition or neural factors within the young group. In studies assessing muscle reflexes, participants possessing a higher proportion of slow-twitch muscle fibers (endurance trained) demonstrated higher sensitivity to mechanical stimuli as compared to participants with a greater proportion of fast-twitch muscle fibers (power trained)^47^. This is in line with findings showing that muscle spindles are more abundant among slow-twitch fibers^48^. Thus, those participants with muscle composition favoring higher slow-twitch proportions may be better suited to respond to relatively static postural perturbations. Conversely, participants with fewer muscle spindles or less sensitive muscle spindles may be less sensitive to slower postural sways. In another postural study where stability was compared between elite wrestlers and untrained, healthy controls, all postural instability variables were increased in the wrestler group^49^. Chronic elite sport training should elicit known modifications to skeletal muscle, inducing changes in size (increased) and muscle fiber composition, and in the case of wrestlers, increased ratios of fast-twitch/slow-twitch fiber areas in the lower and upper limbs compared to controls^50^. Therefore, this previous research supports the notion that participants with ‘better’ muscle composition and tentatively higher fast-twitch muscle proportions perform less well at static balance. Worthy of note, it is likely that our sample of young participants featured various profiles of physical activity, since most were recruited among students in a physical therapy program. With this said, although our data align well with this view, it does not bring further supportive arguments as we did not screen for those regularly participating in strength training or regular sport or utilize fiber type analyses.

Interestingly, muscle composition may have a differing contribution to static as opposed to dynamic balance. Indeed, a greater abundance of fast-twitch muscle fibers appears to be associated with improved reactive balance, where environmental induced perturbations disturb one’s center of mass due to a more rapid kinematic response^51^. Therefore, static and reactive balance stability might be favored by different muscle fiber compositions, with static balance benefitting from a higher proportion of slow-twitch fibers and dynamic balance from fast-twitch fibers.

With regards to the classification performance of conventional and texture-based parameters, our AUC pairwise analysis demonstrated that EI, MT, ASM, and IDM possessed outstanding classification ability^36^. Importantly, although EI featured the highest AUC, it only showed a non-significant trend in outperforming the most promising texture-based parameter (ASM; *p* = 0.075). Considering this and the fact that texture-based parameters are robust across US equipment settings, ASM appears to be a very promising alternative to more conventional EI-based metrics to detect age-related differences in muscle composition in future research studies. However, in contexts where the same US equipment can be used across patients, EI and MT remain the measures of choice for classification purposes.

In our study, differences in GLCM parameters were present in the VL and the BB, with young images corresponding to more homogenous textures, and older images corresponding to more heterogeneous textures, supporting previous work^19^. Indeed, spatially relevant imaging parameters have been used in non-muscle imaging, where diseased tissues possess different traits compared with normal healthy tissues^52–54^. Considering the lack of a single diagnostic criterion for sarcopenia and other muscle disorders, tools that facilitate comparison of results between studies and populations are necessary^55^ and texture-based US analysis appears as a viable option. Already, altered textures have been reported in dynapenia showing its potential application to the characterization of aging muscle^56^.

Another critical piece in aging muscle assessment lies in muscle size. In typical physiological aging, muscle mass decreases. In our study, we identified differences in MT in the thigh, but not in the arm. Previous work has shown that muscle thickness and torque generation in the lower extremity is more influenced by aging compared to the upper extremity^25,26^. It is possible that our lower extremities may respond more robustly to changes in physical activity and/or sedentary time with age, as this disuse of our primary locomotive equipment could selectively result in atrophy in the lower body, while the upper extremity muscle size could be spared due to use in everyday activities. Therefore, the estimation of muscle size of the leg may be more likely to show age-related reductions in size, compared to the arm.

Continuing on the topic of morphology, we found that SAT was not different between groups at the thigh or the arm. This was not the case for EI, where older muscle showed higher EI values in both locations. Together, these results suggest specific changes in fat infiltration in aging, that were independent of anatomical location, and occurred in a muscle appearing to atrophy (VL) and a muscle appearing to maintain its size (BB) across the age groups. Thus, we suggest that EI measures may be independent of muscle size. It should be noted though that it is difficult to determine whether fat infiltration is directly related to aging or to physical inactivity^57^ since the accumulation of intramuscular fat occurs even in young, healthy adults exposed to immobilization^58^, as well as in paretic limbs of stroke survivors^59^, and post-spinal cord injury^60^.

Although the VL, which we studied here, is the muscle often looked at in investigations of sarcopenia or fall risk, due to its antigravity function and importance in ambulation^61^, it is possible that other lower-limb muscles, like the tibialis anterior and triceps surae, possess relevant US features that may predict balance. Inclusion of lower leg muscles, especially in the study of standing balance, may provide insight into muscular contributions that the VL cannot explain. For example, in a previous study, COP displacements were best explained by tibialis anterior muscle activity, as compared to muscles of the thigh^62^. Importantly, several characteristics of the triceps surae and ankle have been reported to play a role in balance, with previous work showing decreased muscle size, strength, activation capacity, and tendon mechanical properties resulted in worse balance^63^. These latter features, although not assessed in our study, should be included in future work to generate a more comprehensive understanding of the factors that impact postural performance. Even within the lower leg, it has been shown that the gastrocnemius and soleus appear to deteriorate at different times and paces^62,64^. Thus, it is clear that future studies of postural control should incorporate these muscles.

Additionally, future research examining whether texture-based approaches are sensitive to exercise or rehabilitation-based interventions will be necessary to determine their efficacy not only as a screening tool but also for clinical follow-ups. This distinction is important to clarify as there has been a lack of consistency in EI measures in response to longer term interventions or during longitudinal clinical studies, whether it be strength training^65^, following stroke^66^, or as a result of immobilization^67^.

Furthermore, it should be noted that postural stability in our study was assessed during a static assessment. Comprehensive analyses on the etiology of falls in the elderly reveals that falls tend to occur during motion^68^, with between 30 and 50% of falls being caused by environmental factors (e.g. poor lighting, slippery or uneven surface). The inability to generate a fast and effective postural correction could very well be influenced by muscle composition and/or muscle fiber type. Thus, future comparisons between such features and dynamic postural stability may hold increased ecological validity.

As a side note, our study was conducted with the Vscan Air, a point-of-care US device. This device is small, completely portable and wireless, relatively cheap compared to other common US equipment, and easily operable by smartphone or tablet. Most musculoskeletal US research is conducted using much larger and expensive units (see, e.g.,^19^). However, our results show that the Vscan Air is capable of detecting age-related differences in morphology, EI, and texture-based parameters and does so in a highly reliable way, as attested by an excellent reproducibility within session according to ICCs. Therefore, geriatric medicine stands to benefit from such a device that poses low barriers for implementation.

## Conclusions

We have shown that muscle composition does not relate to balance performance in older individuals, despite the fact that both deteriorate with aging. This suggests that these two commonly documented fall risk factors are independent, hinging on different aging mechanisms. In addition, our data supports previous findings showing that US-derived texture-based parameters provide robust markers of muscle composition. Therefore, as we seek to understand the mechanisms involved in age-related reductions in mobility and fall risk and develop relevant diagnostics, US imaging and subsequent texture-based analyses should be considered.

### Supplementary Information

Supplementary Information.

## Data Availability

US data and analysis scripts used in this study will be made available upon reasonable request to the corresponding author.
