# Supplementary material for: Age-related changes in ultrasound-assessed muscle composition and postural stability
Source: Sci Rep. 2024 Aug 12;14:18688. doi: 10.1038/s41598-024-69374-8 (PMC11319795; doi:10.1038/s41598-024-69374-8)
Supplement: Supplementary file 1 — Supplementary Information. [file 41598_2024_69374_MOESM1_ESM.pdf]

**Supplementary Materials:**

Supplementary Figure 1 presents the $sdCoP_{ML}$ normalized by height in the medio-lateral direction, hereby referred to as $sdCoP_{ML}$ in both groups and in the four standing balance conditions. A two-way ANOVA applied to that measure revealed a significant effect of condition ($F_{3,263} = 54.786$, $p < 0.0001$, $\eta^2 = 0.391$) and age ($F_{1,263} = 72.231$, $p < 0.0001$, $\eta^2 = 0.220$), and a significant interaction thereof ($F_{3,263} = 5.254$, $p = 0.002$, $\eta^2 = 0.058$). The outcome of post-hoc analyses is presented in Supplementary Figure 1. The ECfoam condition resulted in significantly higher $sdCoP_{ML}$ compared to all other conditions, and the EOfoam condition resulted in higher $sdCoP_{ML}$ compared to the hard conditions. In all conditions, older participants had significantly increased $sdCoP_{ML}$ compared to young participants ($p < 0.005$), with the greatest difference between groups in the ECfoam condition.

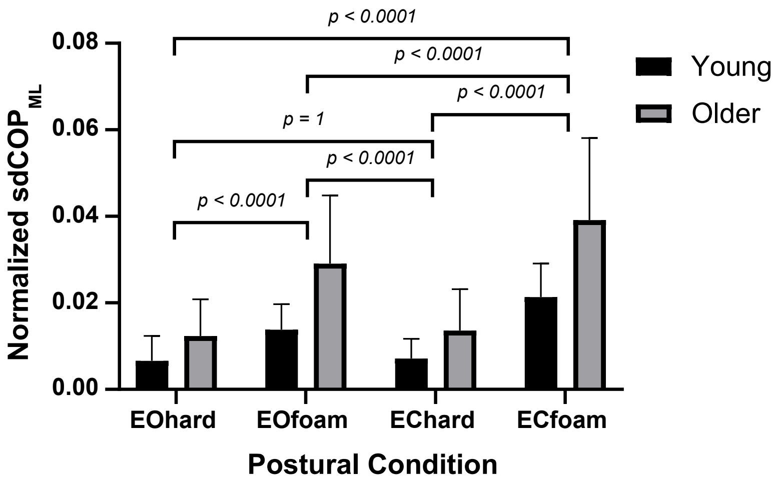


*Supplementary Figure 1. Effect of condition and age on postural stability (medio-lateral). Vertical bars and error bars indicate the mean and standard error of the $sdCOP_{ML}$ for each condition and group (black bars, young group; gray bars, older group). Horizontal lines indicate the p-value for the comparisons of normalized $sdCOP_{ML}$ between conditions.*

A second CCA was conducted on young participants and older participants separately, comparing US parameters and postural stability in the medio-lateral direction across the four conditions. The CCA conducted in both groups did not result in significant relationships (young: $p = 0.519$; old: $p = 0.913$).
